# Cerebrovascular disease and postoperative cognitive-related complications after knee arthroplasty: evidence from a nationwide cohort

**DOI:** 10.1186/s43019-026-00329-1

**Published:** 2026-07-06

**Authors:** Yu Mori, Kunio Tarasawa, Hidetatsu Tanaka, Naoko Mori, Kiyohide Fushimi, Toshimi Aizawa, Kenji Fujimori

**Affiliations:** 1 Department of Orthopaedic Surgery, Tohoku University, Sendai, Japan; 2 Department of Health Administration and Policy, Tohoku University, Sendai, Japan; 3 Department of Radiology, Akita University, Akita, Japan; 4 Department of Health Policy and Informatics, Institute of Science, Tokyo, Tokyo, Japan

**Keywords:** Knee arthroplasty, Cerebrovascular disease, Cognitive-related complications, Propensity score matching, Nationwide database

## Abstract

**Background:**

Patients with a history of cerebrovascular disease may be at an increased risk for postoperative complications following knee arthroplasty; however, previous studies have been limited by small sample sizes and insufficient adjustment for confounding variables. This study aimed to evaluate whether cerebrovascular disease is associated with postoperative complications using a nationwide Japanese database.

**Methods:**

A retrospective cohort study was conducted using Japan’s Diagnosis Procedure Combination database from April 2016 to March 2023. Patients who underwent total knee arthroplasty (TKA) or unicompartmental knee arthroplasty (UKA) were identified, and postoperative complications—including deep vein thrombosis, pulmonary embolism, cerebrovascular events, surgical site infection, cognitive-related complications, and periprosthetic fractures—were evaluated. Cerebrovascular disease was defined using ICD-10 codes I60–I69. Propensity score matching (1:1) was performed using demographics, comorbidities, anesthesia type, and surgical procedure. Multivariate logistic regression was conducted to account for residual confounding.

**Results:**

Among 259,319 eligible patients, 8298 had cerebrovascular disease. After matching, 8269 pairs were analyzed. Before matching, patients with cerebrovascular disease showed higher rates of thromboembolic and infectious complications, longer hospital stays, and greater transfusion volume. After matching, only cognitive-related complications remained significantly more frequent in the cerebrovascular disease group. Cerebrovascular disease was associated with postoperative cognitive-related complications (odds ratio (OR) 1.70; 95% confidence interval (CI) 1.28–2.26; *p* = 0.0003), with a risk difference of 0.62% (95% CI 0.28–0.95). Sensitivity analyses excluding patients with preoperative dementia or cognitive impairment and analyses limited to TKA cases demonstrated directionally consistent findings, although these associations did not reach the prespecified stringent significance threshold.

**Conclusions:**

Cerebrovascular disease does not increase the risk of recurrent cerebrovascular events after knee arthroplasty; however, it elevates the risk of postoperative cognitive-related complications, despite a low absolute incidence. Although the overall incidence was low, this finding may have implications for postoperative recovery and functional outcomes.

**Level of evidence:**

III (retrospective cohort study).

**Supplementary Information:**

The online version contains supplementary material available at 10.1186/s43019-026-00329-1.

## Introduction

Cerebrovascular disease is a major public health concern and remains one of the leading causes of long-term disability worldwide [1, 2]. As life expectancy increases, a growing number of patients with a history of cerebrovascular disease undergo orthopedic procedures, including total knee arthroplasty (TKA) and unicompartmental knee arthroplasty (UKA), to improve mobility and quality of life [3, 4]. However, patients with prior cerebrovascular disease may have unique perioperative risks due to underlying neurological deficits, impaired cerebral autoregulation, and the frequent need for antithrombotic therapy [5, 6].

Previous studies have suggested that patients with a history of cerebrovascular disease may have an increased risk of postoperative complications such as recurrent cerebrovascular events, delirium, prolonged hospitalization, and functional decline after major surgery, including arthroplasty [5, 7–9]. Nevertheless, most of these studies were limited by small sample sizes, single-center designs, or insufficient adjustment for confounding variables. In particular, few investigations have rigorously examined whether the elevated risks observed in patients with cerebrovascular disease can be fully explained by baseline comorbidities, or whether cerebrovascular disease itself contributes to adverse postoperative outcomes [10].

Furthermore, the perioperative management of antithrombotic therapy represents an additional layer of complexity in this patient population. Patients with cerebrovascular disease often require chronic antiplatelet or anticoagulant therapy, which may influence perioperative bleeding, transfusion requirements, and thromboembolic events [11–13]. Despite its clinical relevance, few large-scale studies have comprehensively evaluated the distribution of antithrombotic agents or incorporated antithrombotic therapy into a risk-adjusted comparison of postoperative outcomes in patients with and without cerebrovascular disease and undergoing knee arthroplasty [14, 15].

Given these gaps in the literature, a large national database study with meticulous adjustment for confounding factors—including comorbidities and antithrombotic medication use—is needed to clarify the true postoperative risk profile of patients with cerebrovascular disease who undergo knee arthroplasty. Therefore, the present study used Japan’s nationwide Diagnosis Procedure Combination (DPC) database to compare the incidence of in-hospital postoperative complications between patients with and without cerebrovascular disease undergoing TKA or UKA, and to determine whether cerebrovascular disease contributes to adverse postoperative outcomes after adjustment for demographic, clinical, and pharmacologic factors [16–26]. We evaluated postoperative outcomes including deep vein thrombosis, pulmonary embolism, cerebrovascular events, surgical site infections, cognitive-related complications, and periprosthetic fractures. To minimize confounding, comparisons were conducted between propensity score–matched cohorts based on age, sex, anesthesia type, surgical procedure (TKA, UKA, or simultaneous bilateral surgery), Charlson Comorbidity Index, and relevant comorbidities. We hypothesized that, despite the contemporary widespread use of antithrombotic therapy, patients with cerebrovascular disease would exhibit significantly higher risks of postoperative cerebrovascular events and cognitive-related complications compared with those without cerebrovascular disease.

## Methods

### Study design

This study was conducted in accordance with the ethical principles of the Declaration of Helsinki and was approved by our Institutional Review Board. The dataset was retrospectively extracted from Japan’s nationwide DPC database, as previously described [27, 28]. Informed consent was obtained through an opt-out approach. Upon admission, patients were informed that their clinical data might be used for academic research and were given the option to decline participation. In addition, no personally identifiable information was included in this study. This research was conducted between April 2016 and March 2023 as part of a nationwide survey involving hospitals participating in Japan’s DPC system. During this interval, approximately 1100 hospitals consistently contributed medical records and granted approval for their inclusion in the study.

Patients with cerebrovascular disease were identified using the International Statistical Classification of Diseases and Related Health Problems, 10th Revision (ICD-10) codes I60–I69 recorded at admission. Patients who underwent knee arthroplasty at participating hospitals were included in the analysis, allowing for a comprehensive assessment of contemporary surgical practices and outcomes across Japan. This study focused on individuals who underwent TKA or UKA, with particular emphasis on postoperative complications in patients with cerebrovascular disease compared with those without. The postoperative complications assessed in this study included deep vein thrombosis, pulmonary embolism, cerebrovascular events, surgical site infection, cognitive-related complications, and periprosthetic fractures. Postoperative cognitive-related complications were identified using the following International Classification of Diseases, 10th Revision (ICD-10) codes: F010, F011, F012, F019, F03, F107, G238, G300, G301, G308, G309, G310, and G318 [17, 29]. Preoperative cognitive impairment, including dementia, was incorporated as a covariate in the propensity score model. Postoperative cognitive-related outcomes were defined using relevant ICD-10 codes newly recorded during the index hospitalization after surgery, rather than preoperative diagnoses.

The primary indications for TKA were identified using ICD-10 codes, focusing on knee joint deformities resulting from osteoarthritis and rheumatoid arthritis. Osteoarthritis was classified using ICD-10 codes M170–M175 and M179, while rheumatoid arthritis was identified using codes M0586, M0606, M0686, and M0696. The use of antithrombotic agents was evaluated on the basis of medications administered during the index hospitalization, as recorded in the administrative claims data. Information on chronic preoperative use prior to admission was not available. Antithrombotic medication use was not included in the propensity score model and was analyzed descriptively without adjustment. Patients with and without cerebrovascular disease who underwent knee arthroplasty were identified on the basis of three key criteria: (1) the main diagnosis, (2) the primary reason for hospitalization, and (3) the condition requiring the greatest medical resources. Cases involving bilateral simultaneous procedures were included, whereas patients who underwent revision knee arthroplasty were excluded. Additionally, arthroplasty performed for trauma or bone tumors was excluded from the analysis.

### Propensity score matching

This study compared postoperative complications between patients with cerebrovascular disease and those without who underwent knee arthroplasty. A 1:1 propensity score–matching methodology was used to balance baseline characteristics between the two groups. The analysis accounted for potential confounding variables, including age, sex, body mass index, general anesthesia, type of surgery (TKA or UKA), bilateral simultaneous procedures, and comorbidities such as diabetes, hypertension, ischemic heart disease, chronic renal dysfunction, hyperlipidemia, cognitive impairment, and rheumatic disease. Additional comorbidities were adjusted for using the Charlson Comorbidity Index. The discriminatory ability of the propensity score model was evaluated using the *C*-statistic. Propensity scores were estimated and matched using nearest-neighbor matching without replacement, with a caliper width set to 0.2 times the standard deviation of the propensity score logit, as previously described [30]. This approach yielded a well-balanced cohort of patients with and without cerebrovascular disease, allowing for valid and reliable comparative analyses.

### Sensitivity analysis

To assess the robustness of the primary findings, a sensitivity analysis was conducted using the same covariates applied in the primary propensity score model. Propensity scores were re-estimated, and 1:1 nearest-neighbor matching without replacement was performed with a more stringent caliper width of 0.05 times the standard deviation of the propensity score’s logit. This approach generated an alternative matched cohort, allowing evaluation of whether the associations observed in the primary analysis remained consistent under tighter matching conditions.

Additional sensitivity analyses were performed to further evaluate the robustness of the findings. First, patients with documented preoperative dementia or cognitive impairment were excluded, and propensity score matching was repeated using the same covariates. Second, a separate propensity score-matched analysis was conducted in patients undergoing TKA only, because TKA and UKA differ in surgical invasiveness and complication profiles.

### Statistical analysis

Data are presented as the mean ± standard deviation. Differences in clinical parameters, including length of hospital stay and transfusion volume on the day of surgery and postoperative day 1, between the cerebrovascular disease and non-cerebrovascular disease groups were examined using the standardized mean difference (SMD), *χ*^2^ test, or Student’s *t*-test, both before and after propensity score matching. Postoperative complications were similarly compared between the two groups using these statistical methods in both the unmatched and matched cohorts. Multivariate logistic regression was performed to further evaluate the association between cerebrovascular disease and postoperative complications, while accounting for residual confounding. In this model, additional clinically relevant covariates were incorporated to address factors that might not have been fully balanced through propensity score matching, thereby enabling a more comprehensive assessment of independent risk factors for adverse postoperative outcomes. Given the large sample size, a more stringent threshold for statistical significance was applied. All statistical tests were two-sided, and *p*-values < 0.001 were considered statistically significant. Statistical analyses were performed using JMP version 18 (SAS Institute, Cary, NC).

## Results

**Fig. 1:**
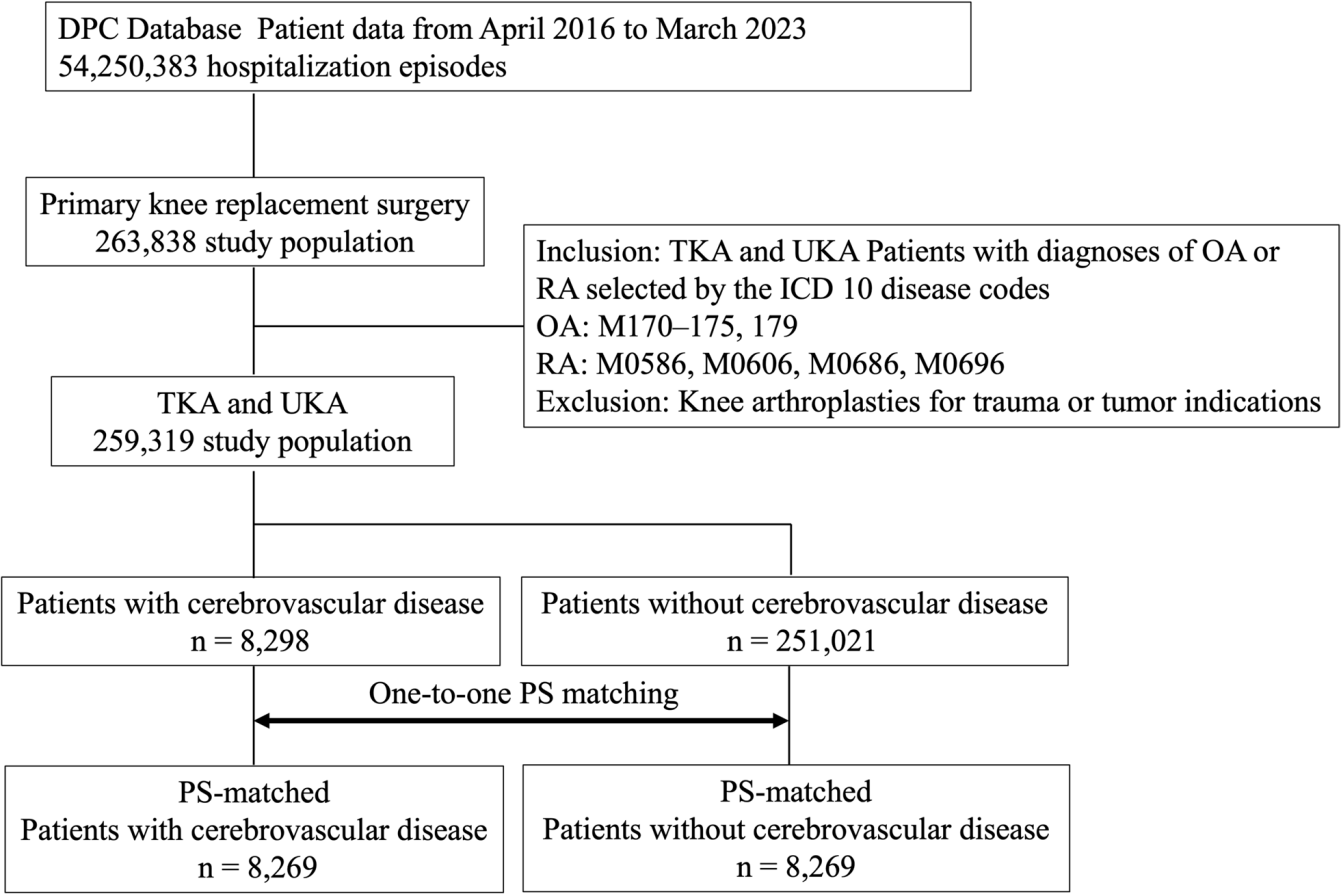
Flow diagram outlining the selection process for patients with and without cerebrovascular disease who underwent knee replacement surgery for osteoarthritis (OA) or rheumatoid arthritis (RA), and the subsequent propensity score (PS) matching procedure. It summarizes the steps used to identify eligible patients from the Diagnosis Procedure Combination (DPC) database and the methodology employed to construct the PS-matched cerebrovascular disease and non-cerebrovascular disease cohorts

Figure 1 illustrates the schematic flow of patient selection. From the dataset covering April 2016 to March 2023, a total of 259,319 patients met the predefined inclusion and exclusion criteria. Of these, 251,021 were assigned to the non-cerebrovascular disease group and 8298 to the cerebrovascular disease group. Following propensity score matching based on variables such as age, sex, Charlson Comorbidity Index, comorbid conditions, type of surgery, type of anesthesia, and simultaneous bilateral procedures, each group included 8269 patients for comparative analysis.

Table 1 summarizes the baseline characteristics of patients with and without cerebrovascular disease who underwent knee arthroplasty. Before propensity score matching, substantial imbalances were observed between the two groups with respect to sex, age, Charlson Comorbidity Index, type 2 diabetes, hypertension, cognitive impairment, and hyperlipidemia, as indicated by standardized mean differences (SMDs) exceeding 0.1. Patients with cerebrovascular disease were more likely to be male, have a higher mean age, and have a greater comorbidity burden reflected by higher Charlson Comorbidity Index scores, and showed higher prevalence rates of type 2 diabetes, hypertension, cognitive impairment, and hyperlipidemia. After 1:1 propensity score matching, most covariates achieved SMD values below 0.1, indicating generally adequate balance between the matched groups. However, the SMD for hypertension remained above 0.1, suggesting incomplete matching for this variable. Although the SMD values for age, Charlson Comorbidity Index, and hyperlipidemia did not exceed the 0.1 threshold, they remained relatively elevated compared with other covariates, implying that balance for these factors may also have been imperfect. Residual imbalance in hypertension was further accounted for in the multivariable regression analysis. The *C*-statistic for the propensity score model was 0.893, indicating satisfactory discrimination.

**Table 1:** Characteristics of patients before and after propensity score matching

	Before PS matching			After PS matching		
	Cerebrovascular disease			Cerebrovascular disease		
	( +)	( −)	SMD	( +)	( −)	SMD
*n*	8298	251,021		8269	8269	
Sex						
Male	2390 (28.8%)	53,936 (21.5%)	0.169	2386 (28.9%)	2377 (28.8%)	0.002
Female	5908 (71.2%)	197,085 (78.5%)		5883 (71.1%)	5892 (71.2%)	
Age	77.2 ± 6.7	74.6 ± 7.8	0.351	77.2 ± 6.6	77.8 ± 6.6	0.098
BMI	25.9 ± 3.9	26.1 ± 4.8	0.032	25.9 ± 4.0	25.9 ± 4.1	0.005
CCI	1.81 ± 1.01	0.65 ± 0.91	1.211	1.81 ± 1.01	1.73 ± 1.17	0.079
Type 2 diabetes	2143 (25.8%)	53,031 (21.1%)	0.111	2145 (25.8%)	2126 (25.7%)	0.002
Hypertension	4623 (55.7%)	86,830 (34.6%)	0.435	4609 (55.7%)	5120 (61.9%)	0.126
Ischemic cardiac disease	618 (7.5%)	14,668 (5.8%)	0.065	618 (7.5%)	654 (7.9%)	0.019
Chronic renal dysfunction	350 (4.2%)	6786 (2.7%)	0.084	350 (4.2%)	321 (3.9%)	0.018
Cognitive impairment	288 (3.5%)	3679 (1.5%)	0.132	287 (3.5%)	302 (3.7%)	0.010
Hyperlipidemia	2532 (30.5%)	48,805 (19.4%)	0.259	2525 (30.5%)	2870 (34.7%)	0.090
Rheumatoid arthritis	208 (2.5%)	9291 (3.7%)	0.069	207 (2.5%)	198 (2.4%)	0.007
General anesthesia	7651 (92.2%)	227,880 (90.8%)	0.051	7627 (92.2%)	7655 (92.6%)	0.013
Surgery						
TKA	7419 (89.4%)	221,176 (88.1%)	0.041	7392 (89.4%)	7447 (90.1%)	0.023
UKA	879 (10.6%)	29,845 (11.9%)		877 (10.6%)	822 (9.9%)	
Bilateral surgery	484 (5.8%)	16,166 (6.4%)	0.025	482 (5.8%)	468 (5.7%)	0.009

One-to-one PS matching was performed

Data are shown as mean ± standard deviation

PS, propensity score; SMD, standard mean difference; BMI, body mass index; CCI, Charlson comorbidity index

Table 2 compares postoperative complication rates between patients with and without cerebrovascular disease. Before propensity score matching, the cerebrovascular disease group exhibited significantly higher incidences of deep vein thrombosis, pneumonia, surgical site infection, cognitive-related complications, and cerebrovascular events. Patients with cerebrovascular disease also had a longer hospital stay and a significantly greater transfusion volume on the day of surgery. After propensity score matching, the incidence of cognitive-related complications remained significantly higher in the cerebrovascular disease group. In addition, the cerebrovascular disease group continued to show a longer hospital stay and a higher transfusion volume on the day of surgery compared with patients without cerebrovascular disease. There were no significant differences in the incidence of other postoperative complications, including cerebrovascular events. Univariate logistic regression analysis demonstrated that cerebrovascular disease was significantly associated with cognitive-related complications, with an odds ratio (OR) of 1.68 (95% confidence interval [CI]: 1.26–2.24, *p* = 0.0003). The risk difference for cognitive-related complications was 0.62% (95% CI: 0.28–0.95).

**Table 2:** Comparison of complications before and after propensity score matching

	Before PS matching			After PS matching			
	Cerebrovascular disease			Cerebrovascular disease			
	( +)	( −)	*p*-Value	( +)	( −)	Odds ratio	*p*-Value
						95% CI	
Deep vein thrombosis	836 (10.1%)	22,308 (8.9%)	0.0002*	835 (10.1%)	754 (9.1%)	1.12 (1.01–1.24)	0.033
Pulmonary embolism	25 (0.3%)	706 (0.3%)	0.74	25 (0.3%)	28 (0.3%)	0.89 (0.52–1.53)	0.68
Pneumonia	36 (0.4%)	436 (0.2%)	< 0.0001*	36 (0.4%)	18 (0.2%)	2.00 (1.14–3.53)	0.014
Cognitive dysfunction	127 (1.5%)	1709 (0.7%)	< 0.0001*	127 (1.5%)	76 (0.9%)	1.68 (1.26–2.24)	0.0003*
Cerebrovascular event	87 (1.0%)	799 (0.3%)	< 0.0001*	87 (1.0%)	100 (1.2%)	0.87 (0.65–1.16)	0.34
Surgical site infection	130 (1.6%)	2844 (1.1%)	0.0003*	129 (1.6%)	137 (1.7%)	0.94 (0.74–1.20)	0.62
Length of hospitalization (days)	31.4 ± 18.5	27.8 ± 15.4	< 0.0001*	31.4 ± 18.5	30.5 ± 16.7	NA	0.0007*
Blood transfusion, day 0 (units)	0.08 ± 0.46	0.05 ± 0.40	< 0.0001*	0.08 ± 0.46	0.09 ± 0.50	NA	0.14
Blood transfusion, day 1 (units)	0.04 ± 0.32	0.03 ± 0.29	0.005	0.04 ± 0.32	0.06 ± 0.41	NA	0.005

One-to-one PS matching was performed

^*^p-Values < 0.001 are considered significant by the *χ*^2^ test and Student’s *t*-test; PS, propensity score; CI, confidence interval; NA, not available

The sensitivity analysis using a more stringent caliper width produced results consistent with the primary analysis, demonstrating significantly higher rates of cognitive-related complications in the cerebrovascular disease group. Additionally, patients with cerebrovascular disease continued to show a longer hospital stay and a greater transfusion volume on the day of surgery (Additional file 1: Table S1).

In the sensitivity analysis excluding patients with documented preoperative dementia or cognitive impairment, postoperative cognitive-related complications remained more frequent in the cerebrovascular disease group, showing a directionally consistent association with the primary analysis; however, the association did not reach the prespecified stringent significance threshold (*p* = 0.003) (Additional file 1: Table S2).

Similarly, in the propensity score-matched TKA-only cohort, postoperative cognitive-related complications remained more frequent in the cerebrovascular disease group, with findings directionally consistent with the primary analysis, although the association did not reach the prespecified stringent significance threshold (*p* = 0.003) (Additional file 1: Table S3).

Table 3 presents the results of the multivariate logistic regression analysis performed to further evaluate the association between cerebrovascular disease and postoperative. Cognitive-related complications, incorporating covariates that were not fully balanced after propensity score matching. An increase of 1 year in age was associated with a higher risk of cognitive-related complications (OR, 1.06; 95% CI 1.04–1.09; *p* < 0.0001), and a one-point increase in the Charlson Comorbidity Index similarly elevated the risk (OR, 1.18; 95% CI 1.05–1.32; *p* = 0.009). Cerebrovascular disease also remained significantly associated with postoperative cognitive-related complications (OR, 1.70; 95% CI 1.28–2.26; *p* = 0.0003). In contrast, hypertension and hyperlipidemia were not significantly associated with cognitive-related complications.

**Table 3:** Multivariate logistic analysis for risk factors for cognitive dysfunction after knee replacement surgery during hospitalization

Variable	Odds ratio (95% CI)	*p*-Value
Age	1.06 (1.04–1.09)	< 0.0001*
CCI	1.18 (1.05–1.32)	0.009
Hypertension	0.82 (0.62–1.09)	0.18
Cerebrovascular disease	1.70 (1.28–2.26)	0.0003*
Hyperlipidemia	0.85 (0.62–1.17)	0.32

^*^p-Values < 0.001 are considered significant by the *χ*^2^ test; CI, confidence interval

Table 4 summarizes the use of antithrombotic agents in patients with and without cerebrovascular disease. Before propensity score matching, substantial differences were observed between the two groups across several antithrombotic medications. Patients with cerebrovascular disease had significantly higher rates of use for aspirin, warfarin, clopidogrel, and apixaban, reflecting differences in baseline clinical characteristics. After propensity score matching, significant differences in the use of several antithrombotic agents persisted. Aspirin and clopidogrel were more commonly prescribed in the cerebrovascular disease group. In contrast, edoxaban and enoxaparin were more frequently used in the non-cerebrovascular disease group. In addition, the use of fondaparinux and warfarin showed no significant differences between the matched groups, and apixaban use did not differ significantly after matching. These findings demonstrate that antithrombotic medication use remained imbalanced between the groups even after matching.

**Table 4:** Comparison of antithrombotic therapies before and after propensity score matching

	Before PS matching			After PS matching		
	Cerebrovascular disease			Cerebrovascular disease		
	( +)	( −)	*p*-Value	( +)	( −)	*p*-Value
Edoxaban	3773 (45.5%)	153,596 (61.2%)	< 0.0001*	3758 (45.5%)	4843 (58.6%)	< 0.0001*
Fondaparinux	173 (2.1%)	5213 (2.1%)	0.96	173 (2.1%)	175 (2.1%)	0.91
Enoxaparin	576 (6.9%)	24,491 (9.8%)	< 0.0001*	572 (6.9%)	733 (8.9%)	< 0.0001*
Aspirin	2821 (34.0%)	19,260 (7.7%)	< 0.0001*	2814 (34.0%)	923 (11.2%)	< 0.0001*
Warfarin	282 (3.4%)	5181 (2.1%)	< 0.0001*	281 (3.4%)	265 (3.2%)	0.49
Clopidogrel	1770 (21.3%)	5381 (2.1%)	< 0.0001*	1767 (21.4%)	321 (3.9%)	< 0.0001*
Apixaban	264 (3.2%)	6175 (2.5%)	< 0.0001*	264 (3.2%)	298 (3.6%)	0.14

One-to-one PS matching was performed

^*^p-Values < 0.001 are considered significant by the *χ*^2^ test; PS, propensity score; SMD, standard mean difference

## Discussion

This nationwide study evaluated postoperative complications in patients with and without cerebrovascular disease undergoing TKA or UKA using a large administrative database. After rigorous adjustment for demographic, clinical, and pharmacologic factors—including antithrombotic medication use—cerebrovascular disease was not associated with a significantly increased risk of postoperative cerebrovascular events. This finding contrasts with prior reports suggesting heightened risk of recurrent cerebrovascular events in this population [5, 7–9]. Several factors may explain this discrepancy. First, the present study incorporated extensive confounding adjustment, including comorbidities and perioperative characteristics that were often not accounted for in previous studies. Second, differences in the use of antithrombotic agents were observed between the groups both before and after matching. However, the direction of these differences varied depending on the specific agent. Therefore, caution is required when interpreting the potential impact of antithrombotic therapy on postoperative outcomes. In addition, differences in study design, including variations in patient selection, outcome definitions, and the use of comprehensive nationwide data with rigorous confounding adjustment, may also contribute to the discrepancy between our findings and those of previous studies.

In contrast, postoperative cognitive-related complications emerged as the only complication that remained significantly associated with cerebrovascular disease after propensity score matching and multivariate analysis. Patients with cerebrovascular disease had a 1.7-fold higher odds of postoperative cognitive-related complications (OR 1.70; 95% CI 1.28–2.26), with an incidence of 1.5% and an absolute risk difference of approximately 0.6%. Although the absolute increase in risk appears modest, the clinical implications are substantial. Postoperative cognitive-related complications can contribute to prolonged hospitalization, delayed rehabilitation, impaired functional recovery, and increased healthcare utilization. Given that knee arthroplasty is performed primarily to improve mobility and quality of life, even small impairments in cognitive status may have a disproportionate impact on postoperative outcomes in vulnerable patients. Although most covariates were well balanced after propensity score matching, hypertension remained slightly imbalanced between the groups. Given that hypertension is associated with both cerebrovascular disease and cognitive outcomes, residual confounding cannot be completely excluded. However, additional multivariable adjustment incorporating hypertension yielded consistent results, supporting the robustness of the observed association. In addition, differences in antithrombotic medication use persisted after matching, and residual confounding related to these variables cannot be excluded. Furthermore, differences in transfusion volume between groups may reflect unmeasured factors such as surgical complexity or perioperative management. Therefore, residual confounding related to these factors cannot be excluded.

Additional sensitivity analyses excluding patients with preoperative dementia or cognitive impairment and analyses limited to patients undergoing TKA demonstrated directionally consistent findings, although these associations did not reach the prespecified stringent significance threshold. These findings suggest that the primary association was not solely attributable to preexisting cognitive disorders or differences between TKA and UKA procedures.

The increased risk of postoperative cognitive complications in patients with cerebrovascular disease may reflect underlying cerebral vulnerability or impaired cerebral autoregulation [31, 32]. However, these mechanisms are speculative and were not directly evaluated in the present study. Therefore, these interpretations should be considered hypothesis-generating rather than conclusive. These mechanisms have been proposed in previous literature but have rarely been demonstrated in large, confounding-adjusted datasets. Further studies are needed to clarify the underlying mechanisms and establish effective preventive strategies.

Importantly, no other postoperative complications—including pneumonia, surgical site infection, thromboembolic events, or periprosthetic fractures—remained significantly different after confounding adjustment. This suggests that, aside from cognitive outcomes, contemporary perioperative care and widespread antithrombotic therapy may attenuate some of the risks previously attributed to cerebrovascular disease.

A major strength of this study is the use of a nationwide administrative database combined with propensity score matching, which enabled effective adjustment for key confounding factors, including age, sex, and comorbidities. The substantial sample size further enhances the statistical robustness and precision of our findings. Nevertheless, several limitations should be acknowledged. First, the study population was restricted to patients undergoing knee arthroplasty in acute care hospitals participating in the DPC system. Consequently, individuals admitted to non-DPC-reported beds—which represent approximately 30% of all general hospital beds—and those treated outside acute care settings were not included [33]. Second, the accuracy of DPC diagnostic classifications could not be independently validated, nor could the clinical severity of comorbid conditions be assessed at the individual patient level. Third, because the outcome was defined using ICD-10 codes from an administrative database, it may include a heterogeneous spectrum of conditions such as delirium, newly recognized cognitive impairment, and dementia-related diagnoses. Therefore, the outcome should be interpreted as postoperative cognitive-related complications rather than strictly defined postoperative cognitive-related complications, and misclassification cannot be completely excluded. To further address this limitation, an additional sensitivity analysis excluding patients with preoperative dementia or cognitive impairment was performed and demonstrated directionally consistent findings. Fourth, although most covariates were well balanced after propensity score matching, some slight residual imbalance remained for certain variables, including hypertension. Given that hypertension is associated with both cerebrovascular disease and cognitive outcomes, residual confounding cannot be completely excluded despite additional multivariable adjustment. Fifth, antithrombotic medication use may represent a potential confounding factor; however, it was not included in the adjustment model, and residual confounding related to these variables cannot be excluded. In addition, the administrative database used in this study does not provide detailed temporal information regarding antithrombotic therapy. Therefore, it was not possible to distinguish between chronic preoperative use and postoperative prophylactic administration. This limitation may affect the interpretation of the relationship between antithrombotic therapy and postoperative outcomes. Sixth, although procedure type (TKA or UKA) was included in the propensity score model, these procedures differ in surgical invasiveness and complication profiles. Therefore, residual confounding related to procedure-specific factors cannot be completely excluded. To further address this concern, an additional propensity-score-matched analysis limited to TKA cases was performed and demonstrated directionally consistent findings. Finally, long-term outcomes such as late infection, periprosthetic fracture, reoperation, or post-discharge mortality were not evaluated.

Despite these limitations, the study has important clinical implications. Our findings indicate that cerebrovascular disease does not inherently increase the risk of recurrent cerebrovascular events after knee arthroplasty, once confounding factors and antithrombotic therapy are accounted for. However, cerebrovascular disease remained significantly associated with postoperative cognitive-related complications, underscoring the need for enhanced perioperative evaluation and tailored management strategies in this vulnerable population. Future large-scale investigations incorporating detailed clinical data, longitudinal follow-up, and targeted interventions to reduce cognitive complications will be essential to further improve outcomes for patients with cerebrovascular disease undergoing knee arthroplasty.

## Conclusions

In this nationwide propensity-score-matched analysis of patients undergoing knee arthroplasty, cerebrovascular disease was not associated with an increased risk of recurrent cerebrovascular events after surgery following adjustment for confounding factors. However, cerebrovascular disease may be associated with increased postoperative cognitive-related complications following knee arthroplasty. Although the overall incidence was low and sensitivity analyses did not reach the prespecified stringent significance threshold, the direction of the associations remained consistent across analyses. This finding may have implications for postoperative recovery and functional outcomes. Further studies incorporating more detailed clinical information and long-term follow-up are needed to clarify underlying mechanisms and downstream functional outcomes.

## Supplementary Information

Additional file1 (DOCX 38 KB)

## Data Availability

Not publicly available owing to institutional regulations but available from the corresponding author upon reasonable request.

## Abbreviations

CI: Confidence interval
DPC: Diagnosis procedure combination
OR: Odds ratio
TKA: Total knee arthroplasty
UKA: Unicompartmental knee arthroplasty
