# Supplementary material for: Cerebrovascular disease and postoperative cognitive-related complications after knee arthroplasty: evidence from a nationwide cohort
Source: Knee Surg Relat Res. 2026 Jul 6;38:26. doi: 10.1186/s43019-026-00329-1 (PMC13340325; doi:10.1186/s43019-026-00329-1)
Supplement: Supplementary file 1 — Additional file1 (DOCX 38 KB) [file 43019_2026_329_MOESM1_ESM.docx]

Supplementary Table 1. Sensitivity analysis performed by altering the caliper width in the propensity score matching

|  | Before PS matching | | | After PS matching | | | |
| --- | --- | --- | --- | --- | --- | --- | --- |
|  | Cerebrovascular disease | | | Cerebrovascular disease | | | |
|  | (+)  n=8298 | (−)  n=251021 | *p*-value | (+)  n=8268 | (−)  n=8268 | Odds ratio | *p*-value |
|  |  |  |  |  |  | 95% CI |  |
| Deep vein thrombosis | 836 (10.1%) | 22308 (8.9%) | 0.0002* | 835 (10.1%) | 758 (9.2%) | 1.11  (1.00-1.23) | 0.042 |
| Pulmonary embolism | 25 (0.3%) | 706 (0.3%) | 0.74 | 25 (0.3%) | 28 (0.3%) | 0.89  (0.52-1.53) | 0.68 |
| Pneumonia | 36 (0.4%) | 436 (0.2%) | < 0.0001* | 36 (0.4%) | 18 (0.2%) | 2.00  (1.14-3.53) | 0.014 |
| Cognitive-related complications | 127 (1.5%) | 1709 (0.7%) | < 0.0001* | 127 (1.5%) | 77 (0.9%) | 1.66  (1.25-2.21) | 0.0004* |
| Cerebrovascular events | 87 (1.0%) | 799 (0.3%) | < 0.0001* | 87 (1.0%) | 101 (1.2%) | 0.86  (0.64-1.15) | 0.30 |
| Surgical site infection | 130 (1.6%) | 2844 (1.1%) | 0.0003* | 129 (1.6%) | 135 (1.6%) | 0.95  (0.75-1.22) | 0.71 |
| Length of hospitalization (days) | 31.4 ± 18.5 | 27.8 ± 15.4 | < 0.0001* | 31.4 ± 18.5 | 30.4 ± 16.6 | NA | 0.0003* |
| Blood transfusion Day 0 (unit) | 0.08 ± 0.46 | 0.05 ± 0.40 | < 0.0001* | 0.08 ± 0.46 | 0.09 ± 0.50 | NA | 0.14 |
| Blood transfusion Day 1 (unit) | 0.04 ± 0.32 | 0.03 ± 0.288 | 0.005 | 0.04 ± 0.32 | 0.06 ± 0.41 | NA | 0.005 |
| One-to-one PS matching was performed. In the sensitivity analysis, propensity score matching was performed using a caliper width of 0.05. | | | | | | | |
| **p*-values of < 0.001 are considered significant by the χ^2^ test and Student’s t-test; PS means propensity score; CI means confidence interval; NA means not available. | | | | | | | |

Supplementary Table 2. Comparison of postoperative complications before and after propensity score matching after exclusion of patients with preoperative dementia or cognitive impairment

|  | Before PS matching | | | After PS matching | | | |
| --- | --- | --- | --- | --- | --- | --- | --- |
|  | Cerebrovascular disease | | | Cerebrovascular disease | | | |
|  | (+)  n=8010 | (−)  n=247342 | *p*-value | (+)  n=7979 | (−)  n=7979 | Odds ratio | *p*-value |
|  |  |  |  |  |  | 95% CI |  |
| Deep vein thrombosis | 807 (10.1%) | 21947 (8.9%) | 0.0002* | 806 (10.1%) | 727 (9.1%) | 1.12  (1.01-1.25) | 0.034 |
| Pulmonary embolism | 24 (0.3%) | 699 (0.3%) | 0.78 | 24 (0.3%) | 39 (0.5%) | 0.61  (0.37-1.02) | 0.06 |
| Pneumonia | 34 (0.4%) | 416 (0.2%) | < 0.0001* | 34 (0.4%) | 27 (0.3%) | 1.26  (0.76-2.09) | 0.37 |
| Cognitive-related complications | 106 (1.3%) | 1567 (0.6%) | < 0.0001* | 106 (1.3%) | 68 (0.8%) | 1.57  (1.15-2.13) | 0.003 |
| Cerebrovascular events | 80 (1.0%) | 767 (0.3%) | < 0.0001* | 80 (1.1%) | 118 (1.5%) | 0.68  (0.51-0.90) | 0.009 |
| Surgical site infection | 123 (1.5%) | 2761 (1.1%) | 0.0009* | 124 (1.7%) | 116 (1.6%) | 1.07  (0.83-1.38) | 0.61 |
| Length of hospitalization (days) | 31.3 ± 18.4 | 27.7 ± 15.3 | < 0.0001* | 31.3 ± 18.4 | 30.2 ± 18.1 | NA | 0.0003* |
| Blood transfusion Day 0 (unit) | 0.08 ± 0.45 | 0.05 ± 0.39 | < 0.0001* | 0.09 ± 0.45 | 0.09 ± 0.52 | NA | 0.11 |
| Blood transfusion Day 1 (unit) | 0.04 ± 0.31 | 0.03 ± 0.28 | 0.012 | 0.04 ± 0.31 | 0.05 ± 0.34 | NA | 0.26 |
| One-to-one PS matching was performed. | | | | | | | |
| **p*-values of < 0.001 are considered significant by the χ^2^ test and Student’s t-test; PS means propensity score; CI means confidence interval; NA means not available. | | | | | | | |

Supplementary Table 3. Comparison of Postoperative Complications Before and After Propensity Score Matching in Patients Undergoing Total Knee Arthroplasty

|  | Before PS matching | | | After PS matching | | | |
| --- | --- | --- | --- | --- | --- | --- | --- |
|  | Cerebrovascular disease | | | Cerebrovascular disease | | | |
|  | (+)  n=7419 | (−)  n=221176 | *p*-value | (+)  n=7388 | (−)  n=7388 | Odds ratio | *p*-value |
|  |  |  |  |  |  | 95% CI |  |
| Deep vein thrombosis | 789 (10.6%) | 20473 (9.3%) | < 0.0001* | 788 (10.7%) | 699 (9.5%) | 1.14  (1.02-1.27) | 0.015 |
| Pulmonary embolism | 23 (0.3%) | 663 (0.3%) | 0.87 | 23 (0.3%) | 31 (0.4%) | 0.74  (0.43-1.27) | 0.27 |
| Pneumonia | 33 (0.4%) | 411 (0.2%) | < 0.0001* | 33 (0.4%) | 24 (0.3%) | 1.38  (0.81-2.33) | 0.23 |
| Cognitive-related complications | 118 (1.6%) | 1595 (0.7%) | < 0.0001* | 118 (1.6%) | 77 (1.0%) | 1.54  (1.15-2.06) | 0.003 |
| Cerebrovascular events | 82 (1.1%) | 730 (0.3%) | < 0.0001* | 82 (1.1%) | 112 (1.5%) | 0.73  (0.55-0.97) | 0.03 |
| Surgical site infection | 125 (1.7%) | 2621 (1.2%) | 0.0002* | 124 (1.7%) | 116 (1.6%) | 1.07  (0.83-1.38) | 0.61 |
| Length of hospitalization (days) | 32.1 ± 18.7 | 28.5 ± 15.8 | < 0.0001* | 32.1 ± 18.7 | 30.9 ± 16.8 | NA | 0.0001* |
| Blood transfusion Day 0 (unit) | 0.09 ± 0.48 | 0.06 ± 0.42 | < 0.0001* | 0.1 ± 0.48 | 0.09 ± 0.54 | NA | 0.38 |
| Blood transfusion Day 1 (unit) | 0.04 ± 0.33 | 0.03 ± 0.29 | 0.009 | 0.04 ± 0.33 | 0.06 ± 0.37 | NA | 0.041 |
| One-to-one PS matching was performed. | | | | | | | |
| **p*-values of < 0.001 are considered significant by the χ^2^ test and Student’s t-test; PS means propensity score; CI means confidence interval; NA means not available. | | | | | | | |
